# Design, Synthesis, and Biological Evaluations of Novel Azothiazoles Based on Thioamide

**DOI:** 10.3390/cimb44070204

**Published:** 2022-07-01

**Authors:** Abdelwahed R. Sayed, Hany Elsawy, Saad Shaaban, Sobhi M. Gomha, Yasair S. Al-Faiyz

**Affiliations:** 1 Department of Chemistry, Faculty of Science, King Faisal University, P.O. Box 380, Hofuf 31982, Saudi Arabia; hmostafa@kfu.edu.sa (H.E.); sibrahim@kfu.edu.sa (S.S.); yalfayz@kfu.edu.sa (Y.S.A.-F.); 2 Department of Chemistry, Faculty of Science, Beni-Suef University, Beni-Suef 62514, Egypt; 3 Department of Chemistry, Faculty of Science, Tanta University, Tanta 31111, Egypt; 4 Organic Chemistry Division, Chemistry Department, Faculty of Science, Mansoura University, Mansoura 35516, Egypt; 5 Department of Chemistry, Faculty of Science, Cairo University, Giza 12613, Egypt; s.m.gomha@gmail.com; 6 Department of Chemistry, Faculty of Science, Islamic University in Almadinah Almonawara, Almadinah Almonawara 42351, Saudi Arabia

**Keywords:** thiazole, hydrazonoyl, carbothioamide, anticancer activities

## Abstract

Herein we studied the preparation of different thiazoles via the reaction of 2-(3,4-dimethoxybenzylidene)hydrazine-1-carbothioamide (**1**) with hydrazonoyl halides under base-catalyzed conditions. The reactions proceed through nucleophilic substitution attack at the halogen atom of the hydrazonoyl halides by the thiol nucleophile to form an *S*-alkylated intermediate. The latter intermediate undergoes cyclization by the loss of water to afford the final products. The structures of the azo compounds were confirmed by FTIR, MS, NMR, and elemental analyses. Indeed, the newly synthesized azo compounds were estimated for their potential anticancer activities by an MTT assay against different human cancer cells, such as lung adenocarcinoma (A549) and colorectal adenocarcinoma (DLD-1). The caspase-3 levels were also estimated using Western blotting and the dual staining technique to evaluate the potency of the titled compounds to promote apoptosis.

## 1. Introduction

Azo dyes have attracted much attention owing to their outstanding biological and physicochemical properties and diverse applications in different aspects of life, such as analytical chemistry, pharmaceutics, cosmetics, painting, and the dyeing industry [1,2,3]. Furthermore, they are easily accessible, relatively stable, and manifest various pharmacological applications (e.g., anticancer, antimicrobial, and antiviral activities) [3,4,5]. Indeed, they are also used in targeted therapy as prodrugs and drug delivery in the case of colitis [6]. Despite being toxic to the environment and humans, many efforts are oriented to modify their structures and improve their biological profiles [7,8,9,10,11].

On the other hand, thiazoles are prepared from the reaction hydrazones with different hydrazonoyl halides [12,13]. They were intensively investigated for their anticancer activities and DNA-binding abilities [14,15]. Moreover, bisthiazoles were prepared from the reaction of bishydrazones with different types of haloketone and were also studied as bioactive skeletons [16]. The goal of this work was to develop novel azothiazoles from the reaction of different hydrazonoyl halides with 2-(3,4-dimethoxybenzylidene)hydrazine-1-carbothioamide (**1**). The final prepared compounds were elucidated by FTIR, MS, NMR, and elemental analyses. Their potential anticancer activities were estimated by MTT assays and their potency to promote apoptosis was estimated with caspase-3 assays.

## 2. Materials and Methods

### 2.1. Synthesis

#### 2.1.1. General Information

The materials were obtained from Fluka and Aldrich. All materials were used without further purification. We checked the melting points by using a Gallen Kamp thermoelectric temperature device. The 1H and 13C NMR spectra were verified in deuterated-dimethyl-sulfoxide with tetramethyl-silane as an internal average via a spectrometer (Varian Gemini 300 MHz). Infrared spectra were investigated via potassium bromide wafer on Pye Unicam, and a Fourier transform infrared spectrophotometer. Elemental microanalyses were done in the micro-analytical laboratory at the University of Cairo, Giza, Egypt.

#### 2.1.2. General Procedure for the Synthesis of Azothiazole Derivatives (**8**–**13**) and (**17**–**19**)

Treatment of 2-(3,4-dihydroxybenzylidene)hydrazine-1-carbothioamide **1** (10 mmol) with other hydrazonoyl chloride **2**–**7** (10 mmol) or bishydrazonyl halides **14**–**16** (5 mmol) was in the presence of an equivalent molar ratio of a basic catalyst such as triethylamine in 15 mL of dioxane. The reaction mixtures were heated under reflux for 6 h, and the solvent was distilled off under reduced pressure. The solid was crystallized from an appropriate solvent and dried at 75 °C for 48 h.

##### 2-(2-(3,4-Dimethoxybenzylidene)hydrazinyl)-5-methyl-4-(phenyldiazenyl)thiazole **8**

Red orange solid (74%); mp. 128–130 °C; IR (KBr): *v* 3188 (NH), 1595 (C=N) cm^−1^. ^1^H NMR (DMSO-*d*_6_): 2. 61 (s, 3H, CH_3_), 3.81 (s, 3H, CH_3_), 3.84 (s, 3H, CH_3_), 7.15–7.77 (m, 8H, ArH), 8.59 (s, 1H, N=CH) and 10.81 (s, 1H, NH) ppm. MS *m/z* (%): 381 (M^+^, 26). Anal. Calcd. for C_19_H_19_N_5_O_2_S (381.13): C, 59.83; H, 5.02; N, 18.36; Found C, 59.71; H, 5.23; N, 18.44%.

##### 2-(2-(3,4-Dimethoxybenzylidene)hydrazinyl)-5-methyl-4-(p-tolyldiazenyl)thiazole **9**

Red orange solid (84%); mp. 140–141 °C; IR (KBr): *v* 3196 (NH), 1609 (C=N) cm^−1^. ^1^H NMR (DMSO-*d*_6_): 2. 31 (s, 3H, CH_3_), 2. 59 (s, 3H, CH_3_), 3.81 (s, 3H, CH_3_), 3.83 (s, 3H, CH_3_), 7.08–7.69 (m, 7H, ArH), 8.91 (s, 1H, N=CH) and 10.74 (s, 1H, NH) ppm. MS *m/z* (%): 395 (M^+^, 21). Anal. Calcd. for C_20_H_21_N_5_O_2_S (395.48): C, 60.74; H, 5.35; N, 17.71; Found C, 60.70; H, 5.64; N, 17.42%.

##### 4-((2-(2-(3,4-Dimethoxybenzylidene)hydrazinyl)-5-methylthiazol-4-yl)diazenyl)benzenesulfonic acid **10**

Red orange solid (77%); mp. 225–227 °C; IR (KBr): *v* 3299 (NH), 1601 (C=N) cm^−1^. ^1^H NMR (DMSO-*d*_6_): 2. 59 (s, 3H, CH_3_), 3.81 (s, 3H, CH_3_), 3.83 (s, 3H, CH_3_), 6.96–7.58 (m, 7H, ArH), 8.41 (s, 1H, N=CH), 8.92 (s, 1H, SO_3_H) and 10.67 (s, 1H, NH) ppm. ^13^C NMR (DMSO-*d*_6_): at 16.47, 55.53, 55.67, 110.04, 113.11, 123.26, 126.43, 126.76, 138.34, 142.28, 143.39, 148.95, 152.05, 160.29 and 171.45 ppm. MS *m/z* (%): 461 (M^+^, 42). Anal. Calcd. for C_19_H_19_N_5_O_5_S_2_ (461.08): C, 49.45; H, 4.15; N, 15.18; Found C, 49.32; H, 4.11; N, 15.29%.

##### 1-(4-((2-(2-(3,4-Dimethoxybenzylidene)hydrazinyl)-5-methylthiazol-4-yl)diazenyl)phenyl)ethan-1-one **11**

Red brown solid (79%); mp. 240–242 °C, IR (KBr): *v* 3322 (NH), 1684 (C=O), 1601 (C=N) cm^−1^. ^1^H NMR (DMSO-*d*_6_): 2.73 (s, 3H, CH_3_), 3.06 (s, 3H, CH_3_), 3.81 (s, 3H, CH_3_), 3.84 (s, 3H, CH_3_), 7.31–7.92 (m, 7H, ArH), 8.61 (s, 1H, N=CH) and 10.73 (s, 1H, NH) ppm. MS *m/z* (%): 423 (M^+^, 22). Anal. Calcd. for C_21_H_21_N_5_O_3_S (423.14): C, 59.56; H, 5.00; N, 16.54; Found C, 59.71; H, 5.12; N, 16.41%.

##### 3-((2-(2-(3,4-Dimethoxybenzylidene)hydrazinyl)-5-methylthiazol-4-yl)diazenyl)phenol **12**

Deep red solid (88%); mp. 200–201 °C, IR (KBr): *v* 3349 (OH), 3262 (NH), 1591 (C=N) cm^−1^. ^1^H NMR (DMSO-*d*_6_): 2. 56 (s, 3H, CH_3_), 3. 81 (s, 3H, CH_3_), 3.84 (s, 3H, CH_3_), 6.72–7.97 (m, 7H, ArH), 8.17 (s, 1H, N=CH), 9.65 (s, 1H, OH), and 11.33 (s, 1H, NH) ppm. ^13^C NMR (DMSO-*d*_6_): at 26.46; 55.47; 55.62; 108.41; 111.16; 115.75; 116.05; 120.18; 122.15; 129.88; 132.06; 133.67; 146.33; 149.10; 150.54; 155.85 and 177.45 ppm. MS *m/z* (%): 397 (M^+^, 33). Anal. Calcd. for C_19_H_19_N_5_O_3_S (397.12): C, 57.42; H, 4.82; N, 17.62; Found C, 57.31; H, 4.70; N, 17.81%.

##### 2-(2-(3,4-Dimethoxybenzylidene)hydrazinyl)-5-methyl-4-((2-nitrophenyl)diazenyl)thiazole **13**

Deep red solid (68%); mp. 220–222 °C, IR (KBr): *v 3*299 (NH), 1605 (C=N) cm^−1^. ^1^H NMR (DMSO-*d*_6_): 2. 59 (s, 3H, CH_3_), 3. 81 (s, 3H, CH_3_), 3.84 (s, 3H, CH_3_), 7.11–7.87 (m, 7H, ArH), 8.65 (s, 1H, N=CH) and 10.99 (s, 1H, NH) ppm. MS *m/z* (%): 426 (M^+^, 23). Anal. Calcd. for C_19_H_18_N_6_O_4_S (426.45): C, 53.51; H, 4.25; N, 19.71; Found C, C, 53.33; H, 4.25; N, 19.94%.

##### 4,4′-((Sulfonylbis(4,1-phenylene))bis(diazene-2,1-diyl))bis(2-(2-(3,4-dimethoxybenzylidene)hydrazinyl)-5-methylthiazole) **17**

Red solid (81%); mp. 235–236 °C, IR (KBr): *v* 3368 (NH), 1593 (C=N) cm^−1^. ^1^H NMR (DMSO-*d*_6_): 2. 59 (s, 6H, 2CH_3_), 3. 81 (s, 6H, 2CH_3_), 3. 83 (s, 6H, 2CH_3_), 7.11–7.88 (m, 14H, ArH), 8.59 (s, 2H, N=CH) and 9.73 (s, 2H, NH) ppm. ^13^C NMR (DMSO-*d*_6_): at 25.57, 55.52, 55.67, 109.98, 111.64, 123.38, 128.17, 128.93, 129.32, 133.82, 135.14, 140.74, 147.98, 148.95, 152.09, 160.48 and 177.69. MS *m/z* (%): 824 (M^+^, 17). Anal. Calcd. for C_38_H_36_N_10_O_6_S_3_ (824.20): C, 55.33; H, 4.40; N, 16.98; Found C, 55.27; H, 4.51; N, 16.79%.

##### 1,4-Bis((2-(2-(3,4-dimethoxybenzylidene)hydrazinyl)-5-methylthiazol-4-yl)diazenyl)benzene **18**

Deep red solid (79%); mp. > 300 °C, IR (KBr): *v* 3252 (NH), 1601 (C=N) cm^−1^. ^1^H NMR (DMSO-*d*_6_): 2. 61 (s, 6H, 2CH_3_), 3.81 (s, 6H, 2CH_3_), 3.85 (s, 6H, 2CH_3_), 7.12–7.86 (m, 10H, ArH), 8.61 (s, 2H, N=CH) and 10.71 (s, 2H, NH) ppm. MS *m/z* (%): 684 (M^+^, 28). Anal. Calcd. for C_32_H_32_N_10_O_4_S_2_ (684.20): C, 56.13; H, 4.71; N, 20.45; Found C, 56.21; H, 4.56; N, 20.59%.

##### 1,3-Bis((2-(2-(3,4-dimethoxybenzylidene)hydrazinyl)-5-methylthiazol-4-yl)diazenyl)benzene **19**

Deep red solid (80%); mp. > 300 °C, IR (KBr): *v* 3196 (NH), 1593 (C=N) cm^−1^. ^1^H NMR (DMSO-*d*_6_): 2.62 (s, 6H, 2CH_3_), 3.82 (s, 6H, 2CH_3_), 3.85 (s, 6H, 2CH_3_), 7.01–7.48 (m, 10H, ArH), 8.59 (s, 2H, N=CH) and 10.71 (s, 2H, NH) ppm. MS *m/z* (%): 684 (M^+^, 34). Anal. Calcd. for C_32_H_32_N_10_O_4_S_2_ (684.20): C, 56.13; H, 4.71; N, 20.45; Found C, 56.29; H, 4.95; N, 20.24%.

### 2.2. Biological Evaluations

#### 2.2.1. Cell Culture and MTT Assay

The colon cancer cell lines (DLD-1) and non-small lung cancer cell lines (A549) were plated separately using 96-well plates with a concentration of 1 × 10^4^ cells/well in DMEM media with 1× Antibiotic Antimycotic Solution and 10% fetal bovine serum (Himedia, Mumbai, India) in a CO_2_ incubator at 37 °C with 5% CO_2_. First, the cells were washed with 200 μL of 1× PBS, and then the cells were treated with Samples **1**–**11** (10–100 µM/mL) with various test concentrations of a compound in serum-free media and incubated for 24 h. The medium was aspirated from cells at the end of the treatment period. Next, 0.5 mg/mL MTT prepared in 1× PBS was added and incubated at 37 °C for 4 h using a CO_2_ incubator. After incubation, the medium containing MTT was discarded from the cells and washed using 200 μL of PBS. The formed crystals were dissolved with 100 μL of DMSO and thoroughly mixed. The development of color intensity was evaluated at 570 nm. The formazan dye turns to purple-blue color. The absorbance was measured at 570 nm using a microplate reader.

#### 2.2.2. Immunoblotting

After treatment with different samples on the DLD-1 and A549 cell lines for 24 h, cells were harvested using trypsin and washed twice with ice-cold PBS. For immunoblotting analysis, cells were lysed in RIPA buffer (Sana crus, Biotech, Dallas, TX, USA) for 14 min on ice, and then centrifuged at 8000× *g* for 15 min at 4 °C. Supernatants were collected and estimated using a Bradford assay. A total of 250 μg protein was denatured at 92 °C for 7 min. Equal amounts of protein were then separated on SDS-PAGE gels and transferred to PVDF membranes. Membranes were blocked with 5% nonfat milk at room temperature for 30 h and incubated with primary antibodies (Caspase-3 (1:2000), Cell Signaling Technology (CST; Beverly, MA, USA) overnight at 4 °C, and then with a horseradish peroxidase-conjugated secondary antibody at room temperature for 1 h. Protein bands were visualized by enhanced chemiluminescence (Licor analyzer). B-actin was used as an internal control.

#### 2.2.3. DUAL Stanning

Dual AO/EB fluorescent staining: After 12 h the treated cells were obtained in 12-well plates, human colon cancer cells and lung cancer cells (DLD-1 and A549), respectively, at the logarithmic growth phase, and cells were washed using ice-cold PBS. Dual fluorescent staining solution (1 μL) containing 100 μg/mL AO and 100 μg/mL EB (AO/EB, Sigma, St. Louis, MO, USA) was added to each suspension, and excess stains were removed. The morphology of apoptotic cells was examined, and 100 cells were counted within 20 min using a fluorescent microscope (Leica, Italy). The dual acridine orange/ethidium bromide (AO/EB) staining method was repeated three times at least. The fluorescence was recorded by Image J software, and values are expressed as fold changes.

Statistics: All statistical analyses were performed using the GraphPad 14.0 software for Windows. A *p*-value of <0.05 was considered statistically significant.

## 3. Results

### 3.1. Chemistry

**Scheme 1:**
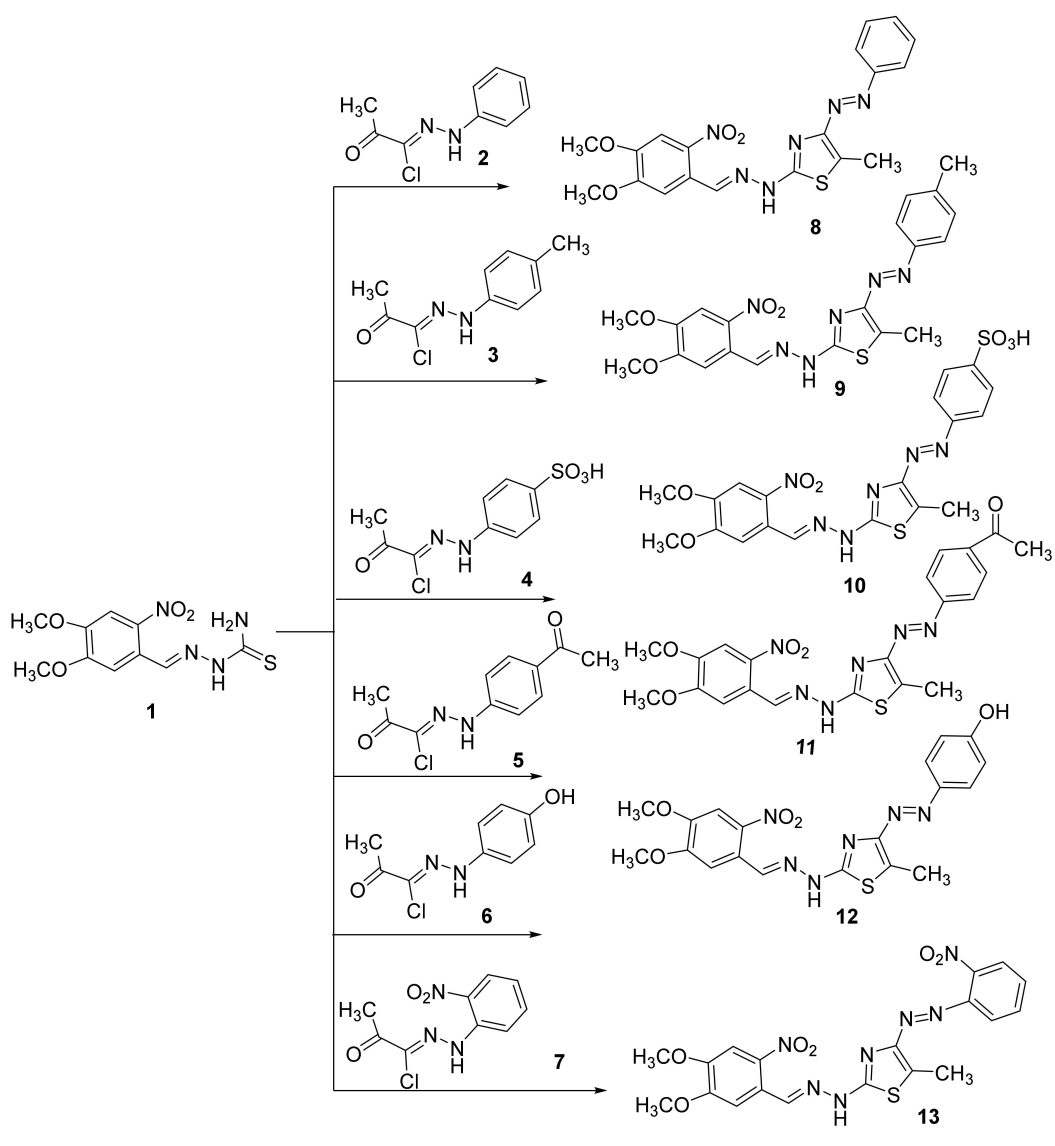
Synthesis of monoazothiazoles **8**–**13.**

Recently, we reported several hydrazonoyl halides and their utility in synthesizing several heterocyclics [17,18,19,20,21,22,23,24]. Herein we focused on developing novel mono and bisazothiazoles. The reaction of 2-(3,4-dihydroxybenzylidene)hydrazine-1-carbothioamide (**1**) with α-haloketohydrazonoyl halides **2**–**7** in a suitable solvent in the presence of base catalyst under heating afforded the corresponding azothiazoles **8**–**13** (Scheme 1). The explanation of the reaction can be through a nucleophilic substitution reaction that starts with the halogen atom from hydrazonoyl halides being replaced by the thiol nucleophile of 2-(3,4-dimethoxybenzylidene)hydrazine-1-carbothioamide to form an *S*-alkylated intermediate. This intermediate could undergo cyclization carried out via nucleophilic addition followed by the loss of water, which would afford the final products. All reactions are given in every example, one product, as observed by TLC. Interestingly, all compounds were prepared in good yields (up to 88%). The final products of the isolated thiazoles **8**–**13** were elucidated using elemental analysis and different spectral technics. For instance, the IR spectrum of the synthesized thiazoles **8**–**13** displayed an absorption peak near 3188–3299 cm^−1^ for the NH.

Furthermore, we found in the final products **8**–**13** disappearance of C=O, which is present in the starting compounds **2**–**7**. All thiazoles **8**–**13** showed the expected molecular ion peaks at the correct *m*/*z* values. Additionally, ^1^H NMR spectra of **8**–**13** showed all protons as expected from the proposed structure, as shown in Scheme 1. The ^13^C NMR for the final products could not be obtained due to poor solubility of most of the compounds, except for compounds **10** and **12** (see experimental part).

**Scheme 2:**
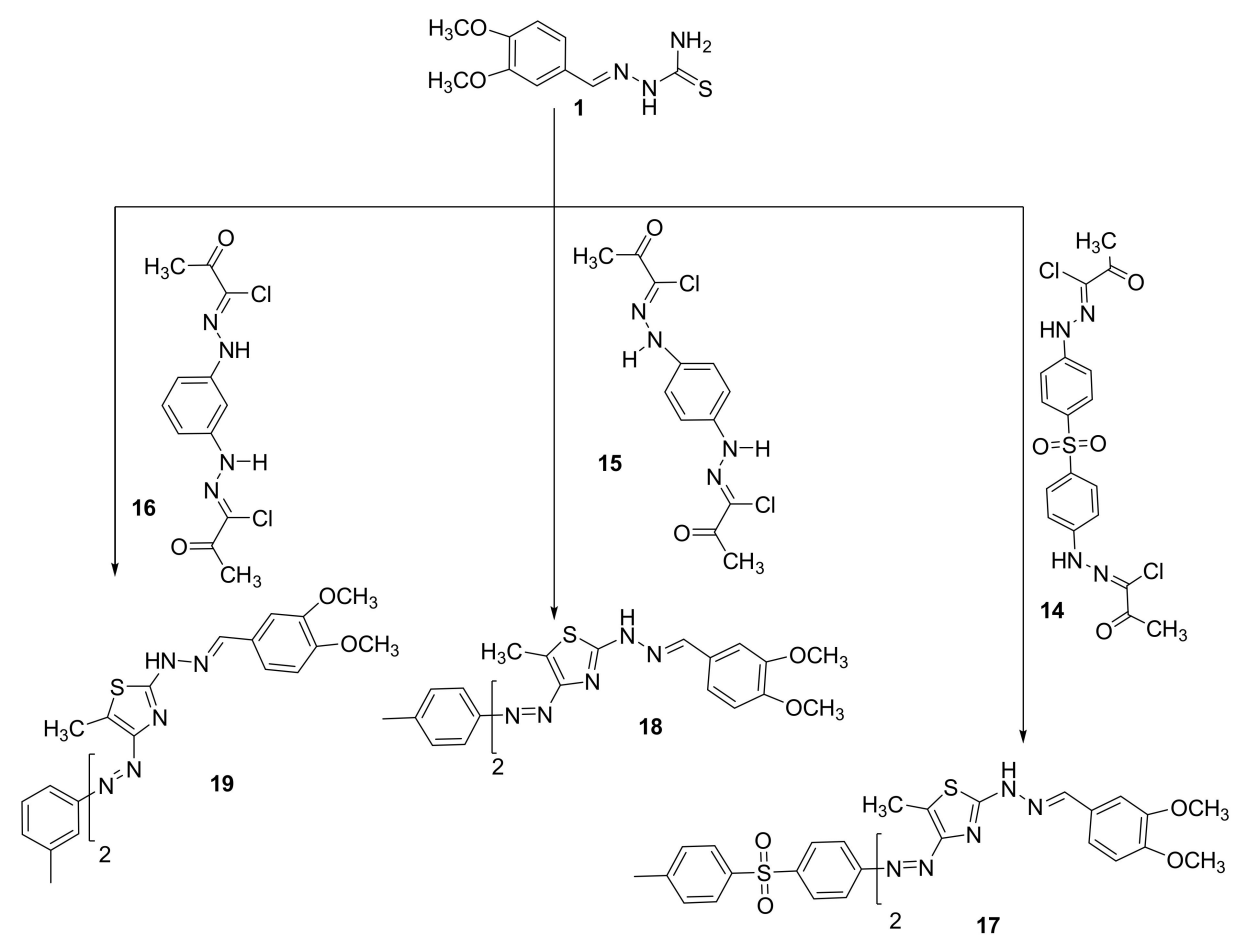
Synthesis of bisazothiazoles **17**–**19.**

The bisazothiazoles were also prepared from the reaction of 2-(4,5-dimethoxy-2-nitrobenzylidene)hydrazine-1-carbothioamide (**1**) with bishydrazonoyl chloride **14**–**16** in Et_3_N/dioxane (Scheme 2). The reactions proceeded through nucleophilic substitution reaction that starts with the halogen atom from bishydrazonoyl halides being replaced by the thiol nucleophile of 2-(3,4-dimethoxybenzylidene)hydrazine-1-carbothioamide (**1**) to form an *S*-alkylated intermediate. This intermediate could undergo cyclization carried out via nucleophilic addition followed by the loss of water, which would afford the final products **17**–**19**, as described in Scheme 2. The novel bisazothiazoles were characterized according by different spectral analysis. For example, the IR spectrum of the synthesized thiazoles **17**–**19** displayed an absorption peak near 3196–3368 cm^−1^ for the NH. Furthermore, we found in the final products **17**–**19** the disappearance of C=O, which is present in the start compounds **2**–**6**. All thiazoles **17**–**19** have shown the expected molecular ion peaks at the foreseeable *m*/*z* values. Additionally, in the ^1^H NMR spectra of **17**–**19** are shown all the protons that were tailored with the proposed structure as shown in Scheme 2. ^13^CNMR for the final products could not appear due to poor solubility, except for compound **17** (see experimental part).

### 3.2. Biology

#### 3.2.1. Cytotoxicity

The azo group is a common functionality in many interesting organic compounds with different applications (e.g., painting, ink, staining of biological systems, and LCD) and potential medicinal activities (e.g., antitumor and antimicrobial) [25,26,27,28]. Furthermore, the anticancer activities of the azo compounds have been reported in numerous reports due to their potency to intercalate with RNA and/or DNA, as well as the inhibition of various proteins [3,4,5,11]. In this regard, the antitumor activities of the novel azo compounds were assessed against the lung A549 and colorectal DLD-1 adenocarcinomas via an MTT assay, and the IC_50_ values are shown in Table 1.

In general, most of the azo compounds showed superior cytotoxicity to the DLD-1 compared to the A549. In the case of DLD-1, azo agents **8** and **11** were the most cytotoxic, with IC_50s_ of 26 ± 2.7 and 21 ± 1.6 µM, respectively, whereas compounds **12** and **13** showed moderate anticancer activity, with IC_50s_ of 32 ± 2.9 and 35 ± 4.21 µM, respectively. In the case of A549 cells, azo compound **12** was the most cytotoxic, with an IC_50_ of 23 ± 2.9 µM. Furthermore, compounds **8**, **11**, and **13** were the least active, with IC_50s_ of 42 ± 3.7, 40 ± 3.83, and 32 ± 4.1 µM, respectively. Collectively, the most cytotoxic compounds were further selected for further biological investigations.

#### 3.2.2. Caspase Activity

**Figure 1:**
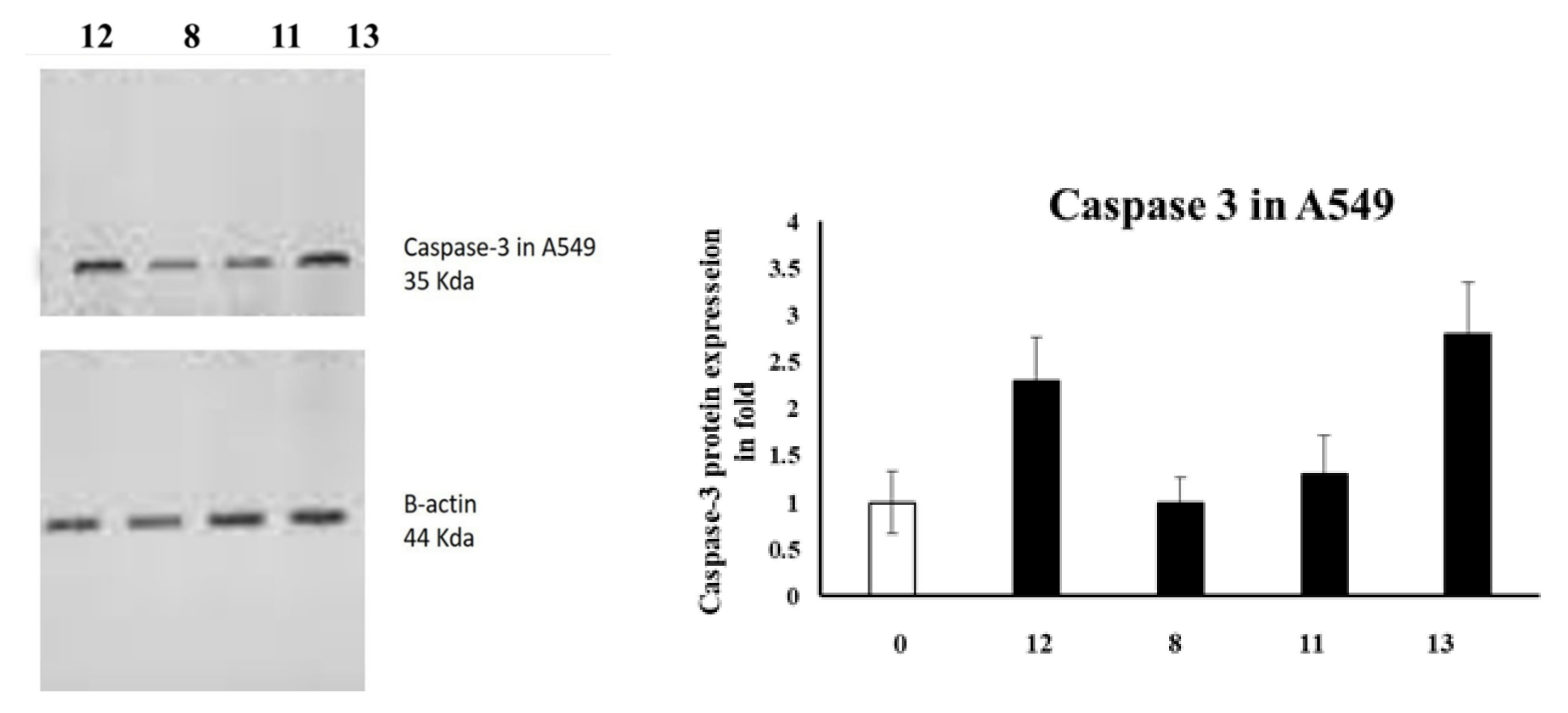

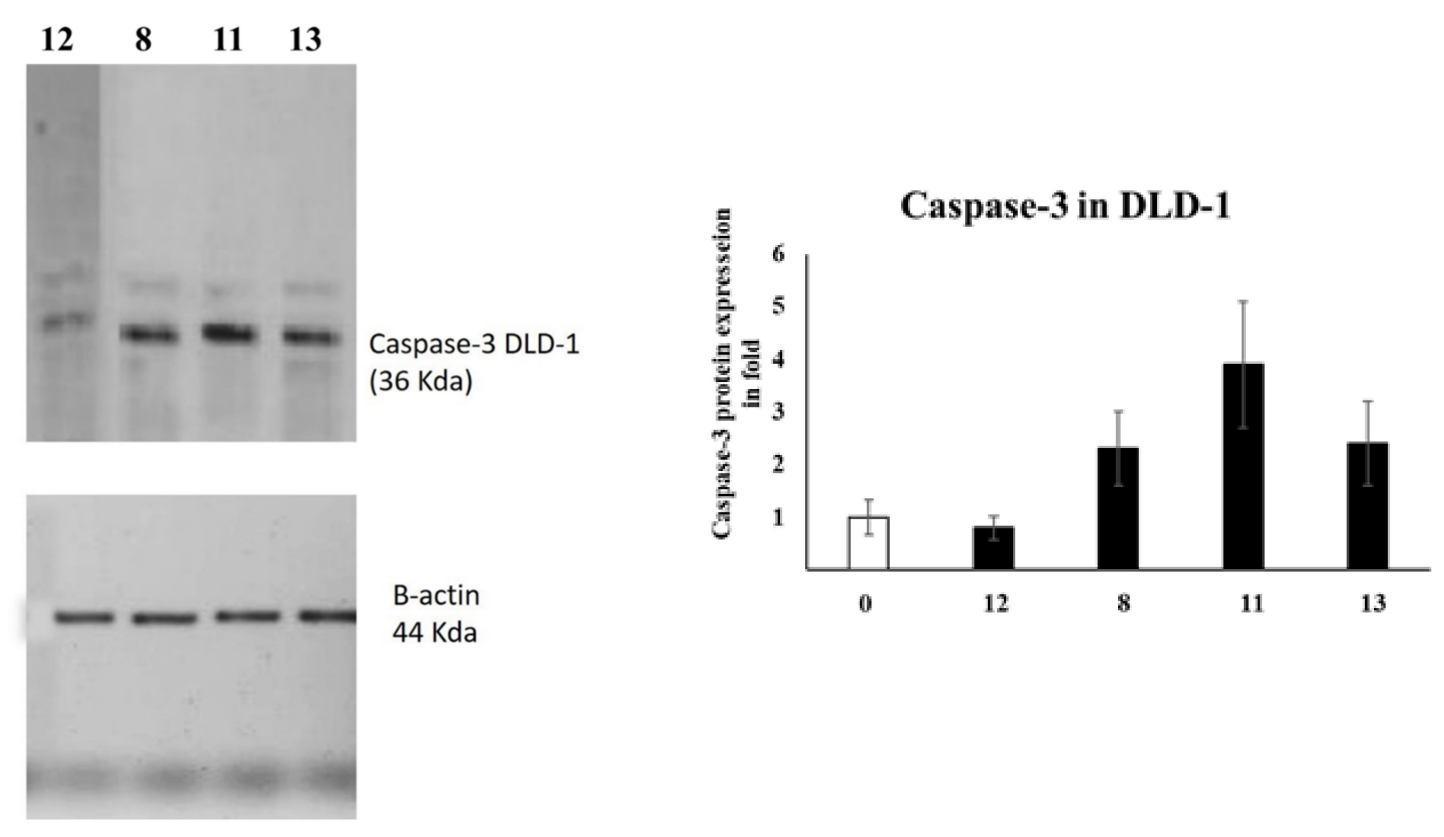
The levels of Caspase-3 in A5941 and DLD-1 cells of various groups were examined by Western blotting.

Caspases are among the proteases involved in apoptosis induction [28]. In particular, caspase-3 is an effector caspase enrolled in the apoptosis execution final stage [4,29]. Accordingly, caspase-3 activation is routinely estimated by medicinal chemists to evaluate the apoptosis as an indication [30,31]. Therefore, the caspase-3 levels were assessed in A549 and DLD-1 cells employing Western blotting, as presented in Figure 1.

In the case of DLD-1 cells, azo compound **11** was the most active and upregulated caspase-3 expression by 4-fold compared to the negative (untreated) control. Furthermore, compound **13** could also activate caspase-3 expression up to 3-fold compared to the negative control.

In the case of A549 cells, azo compound **13** upregulated caspase-3 expression by 3.5-fold compared to the untreated control, whereas azo compound **12** was only able to activate caspase-3 expression up to 2.5-fold compared to the negative control.

To conclude, our experiments showed that the caspase-3 levels were activated in DLD-1 and A549 cells, which might promote apoptosis and lead to cell death.

#### 3.2.3. Dual AO/EB Fluorescent Staining

**Figure 2:**
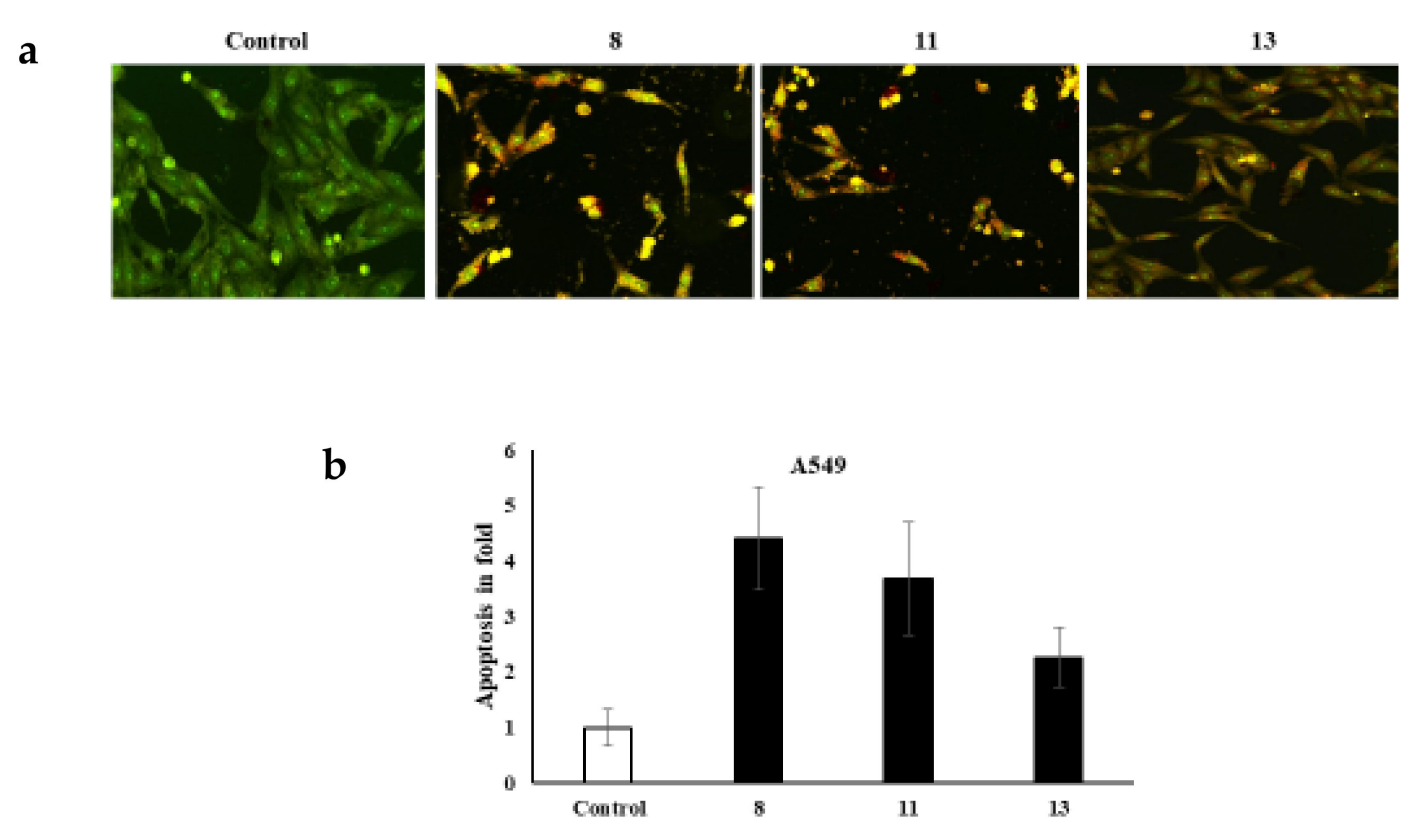
(**a**) dual staining of A549 cells via the fluorescent homidium bromide and acridine orange dyes; (**b**) apoptosis in fold.

**Figure 3:**
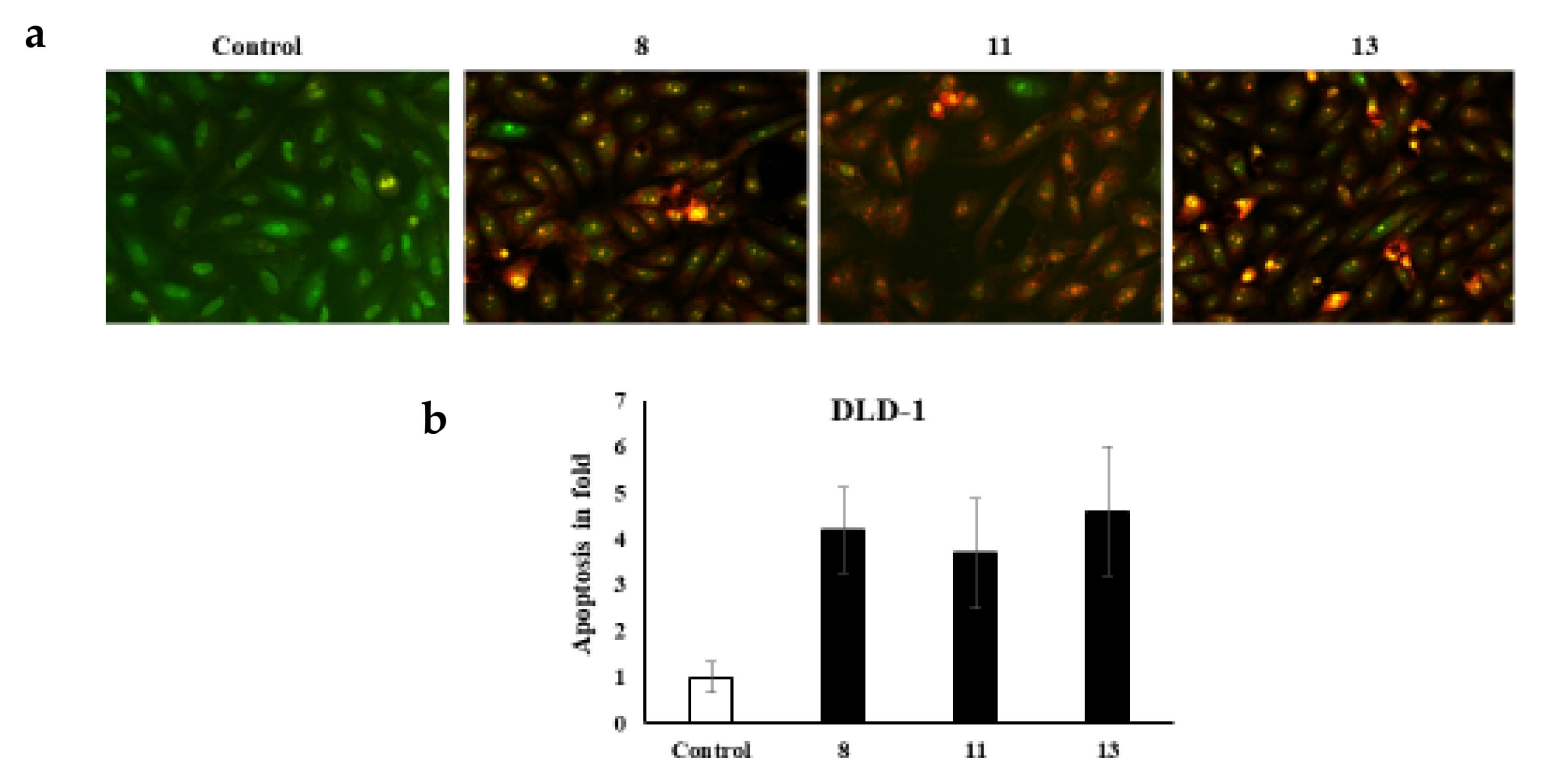
(**a**) dual staining of DLD-1cells via the fluorescent homidium bromide and acridine orange dyes; (**b**) apoptosis in fold.

It is worth noting that various cell morphology alterations accompany apoptosis. Such hallmarks include the formation of cytoplasmic blebs, chromatin condensation, and fragmentation of the nucleus [32,33,34]. Additionally, the shape of the cell turns to be irregular and associated with the formation of apoptotic bodies of DNA fragments as well as the size of the nucleus [32,35,36,37,38,39,40,41]. The novel azo dyes’ anticancer properties and apoptosis induction were further evaluated using a dual staining technique via the fluorescent homidium bromide and acridine orange dyes. It is worth noting that the dual AO/EB fluorescent staining technique revealed the differences between necrotic and apoptotic cells. In this regard, it is obvious that the novel azoic dyes promote different cell morphology alterations, as shown in Figure 2 and Figure 3.

## 4. Conclusions

This article described the design, synthesis, and biological studies of a novel series of azothiazoles based on hydrazonoyl halides. The explanation of the reaction can be through a nucleophilic substitution reaction that starts with the halogen atom from hydrazonoyl halides being replaced by the thiol nucleophile of 2-(3,4-dimethoxybenzylidene)hydrazine-1-carbothioamide (**1**) to form an *S*-alkylated intermediate. This intermediate could undergo cyclization carried out via nucleophilic addition followed by the loss of water, which would afford a novel series of azothiazoles. These reactions are provided the desired products in excellent yields. FTIR, MS, and NMR spectra elucidated structures of the final products. The new series of benzothiazoles were investigated for their potential anticancer activities and evaluated the potency of the titled compounds to promote apoptosis.

## Figures and Tables

**Table 1:** Effect of the synthesized compounds on the viability of human colorectal adenocarcinoma (DLD-1) and human adenocarcinoma(A549) cells.

Compounds	IC_50_ (µM) ^a^	
	A549	DLD-1
**Cisplatin**	6 ± 1.2	7.3 ± 2.6
**8**	42 ± 3.7	26 ± 2.7
**9**	- ^b^	- ^b^
**10**	- ^b^	- ^b^
**11**	40 ± 3.83	21 ± 1.6
**12**	23 ± 2.9	32 ± 2.9
**13**	32 ± 4.1	35 ± 4.21
**17**	- ^b^	- ^b^
**18**	- ^b^	43
**19**	- ^b^	- ^b^

^a^ Cytotoxicity was measured after forty-eight hours treatment of DLD-1 and A549 cells with serial concentrations of the newly synthesized compounds using an MTT assay. ^b^ No proliferation inhibition (IC_50_ ≥ 50) was observed at the tested concentration range.

## Data Availability

The raw/processed data generated in this work are available upon request from the corresponding author.
